# Amniotic fluid disorders and the effects on prenatal outcome: a retrospective cohort study

**DOI:** 10.1186/s12884-021-03549-3

**Published:** 2021-01-22

**Authors:** H. Bakhsh, H. Alenizy, S. Alenazi, S. Alnasser, N. Alanazi, M. Alsowinea, L. Alharbi, B. Alfaifi

**Affiliations:** 1 Clinical Sciences Department, College of Medicine, Princess Nourah bint Abdulrahman University, Riyadh, Saudi Arabia; 2 Department of Obstetrics & Gynecology, King Abdullah bin Abdulaziz University Hospital, Riyadh, Saudi Arabia; 3 College of Medicine, Princess Nourah bint Abdulrahman University, Riyadh, Saudi Arabia

**Keywords:** Amniotic fluid, Disorders, Parental outcome, Saudi Arabia

## Abstract

**Background:**

The amniotic fluid is a protective liquid present in the amniotic sac. Two types of amniotic fluid disorders have been identified. First refers to polyhydramnios, which is an immoderate volume of amniotic fluid with an Amniotic Fluid Index (AFI) greater than 24 cm. Second includes oligohydramnios, which refers to decreased AFI i.e., less than 5 cm. This study aims to; a) identify the maternal risk factors associated with amniotic fluid disorders, b) assess the effect of amniotic fluid disorders on maternal and fetal outcome c) examine the mode of delivery in pregnancy complicated with amniotic fluid disorders.

**Methods:**

A comparative retrospective cohort study design is followed. Sample of 497 pregnant women who received care at King Abdullah bin Abdul-Aziz University Hospital (KAAUH) between January 2017 to October 2019 was included. Data were collected from electronic medical reports, and was analyzed using descriptive statistics. Association of qualitative variables was conducted by Chi-square test, where *p*-value < 0.05 was considered statistically significant.

**Results:**

Among the collected data, 2.8% of the cases had polyhydramnios and 11.7% patients had oligohydramnios. One case of still born was identified. A statically significant association was found between polyhydramnios and late term deliveries (*P* = 0.005) and cesarean section (CS) rates (*P* = 0.008). The rate of term deliveries was equal in normal AFI and oligohydramnios group (*P* = 0.005). Oligohydramnios was mostly associated with vaginal deliveries (*P* = 0.008). Oligohydramnios and polyhydramnios were found to be associated with diabetes mellitus patients (*P* = 0.005), and polyhydramnios with gestational diabetes patients (*P* = 0.052). Other maternal chronic diseases showed no effect on amniotic fluid index, although it might cause other risks on the fetus.

**Conclusion:**

Diabetes mellitus and gestational diabetes are the most important maternal risk factors that can cause amniotic fluid disorders. Maternal and fetal outcome data showed that oligohydramnios associated with gestational age at term and low neonatal birth weight with high rates of vaginal deliveries, while polyhydramnios associated with gestational age at late term and high birth weight with higher rates of CS.

**Supplementary Information:**

The online version contains supplementary material available at 10.1186/s12884-021-03549-3.

## Background

The amniotic fluid is the protective liquid present in the amniotic sac, and mainly composed of water and solids, including proteins, carbohydrates, lipids and phospholipids, enzymes, hormones and chemical substances urea, uric acid, creatinine, electrolytes. It is developed from the membrane plasma by the development of fetus and serves as a protective cushion for the growing fetus. It has antibacterial properties to protect the growing fetus from infection, also it facilitates the exchange of nutrients, water and biochemical products between mother and fetus. Different types of fetal cells are further present which includes; skin, respiratory, intestinal, urinary tract and stem cells, hair and blood cells, all of which aid in the growth of fetus.

Amniotic Fluid Volume (AFV) does not change significantly from day to day, but generally it increases with the growth of fetus reaching a peak at 34 weeks of gestation (over 800 mL), after which it may start to decrease [1]. However, in certain cases Amniotic Fluid Index (AFI) of the amniotic fluid volume is greater than 24 cm, the phenomenon is referred as polyhydramnios [2]. Risk factors of polyhydramnios includes: maternal diabetes, fetal heart failure, abnormal swallowing and congenital infection [3]. The degree of increase in the AFV is directly associated with the increase in adverse risk factors on mother and fetus prenatally. Some of these prenatally outcomes include preterm birth, cesarean delivery [4], placental abruption, fetal malposition, macrosomia, umbilical cord prolapse and maternal respiratory compromise [2].

In contrast, AFI of the amniotic volume less than 5 cm is referred as oligohydramnios [5]. Risk factors of oligohydramnios includes: premature rupture of membranes, intrauterine growth restriction and birth defects [3]. Oligohydramnios impede normal fetal movement, retarded fetal growth and development leading towards fetal deformities, umbilical cord compression, while in some cases death may happen depending on gestational age. In the first trimester decrease in AFI is an ominous finding, the fetus usually undergo abortion. However, in the second trimester the prognosis majorly depends mostly on the etiology, borderline/low normal amniotic fluid volume which generally have a good prognosis, on the other hand severe oligohydramnios often lead to fetal death [6]. Whereas, in the third trimester many cases of oligohydramnios are idiopathic. The occurrence of a threatening fetal outcome is related to the umbilical cord compression, utro-placental insufficiency and meconium aspiration [5].

Amniotic Fluid Volume (AFV) reflects the state of pregnancy and possible adverse complications and outcome, due to which assessment of AFV has been part of every second and third trimester ultrasound examination. There are three standard methods for AFV assessment, first includes qualitative and other two are semiquantitative. Qualitative assessment is the first step in which the ultrasonographer subjectively report if the pregnancy is normal, oligo or polyhydramnios. With experienced examiners, the quality of the test can be equal to the semiquantitative tests [single deepest pocket (SDP), amniotic fluid index (AFI)] which are performed when there is an abnormality found in the qualitative test, specifically among patients with increased risk of pregnancy complications and patients in third trimester. Both SDP and AFI are good indicators for normal AFV. However, AFI overly diagnose oligohydramnios, and SDP overly diagnose polyhydramnios.

Due to the observed increase in cases of poly and oligohydramnios, this study estimated the effect of amniotic fluid disorders on prenatal outcome and reassess the reasons associated with it in Saudi Arabia. Besides, the relationship between maternal demographics and its effect on amniotic fluid disorders in Saudi Arabia are further observed.

### Specific objectives

1. To identify the maternal risk factors associated with amniotic fluid disorders.
2. To assess the effect of amniotic fluid disorders on maternal and fetal outcome.
3. To identify the mode of delivery in pregnancy complicated with amniotic fluid disorders.

## Methods

This study followed a comparative retrospective cohort study design. Patients who received care at a secondary hospital i.e., King Abdullah Bin Abdul-Aziz University Hospital (KKAUH) in its obstetrics & gynecology department between January 2017–October 2019 were included. The study was approved by the institutional review board in Princess Nourah bint Abdulrahman University’s (PNU) collage of medicine.

The electronic medical reports of normal and abnormal obstetrical ultrasound examinations reviewed during the study period were searched. These ultrasounds were taken as part of routine check-up or when prescribed by the consulted physician. Patients were included in the cohort, if they had at least one scan conducted by expert radiologists in our ultrasound unit.

A formulated data collection sheet was filled from each patient file without any intervention. Which included the following: demographic data, past medical history, past surgical history, obstetrics & gynecology history, intraoperative complications, postoperative complications and neonatal outcomes. Neonatal outcomes encompass living status, gender, APGAR score, birth weight, admission of baby and congenital malformations. The cohort was stratified into three groups based on AFI: normal AFI, polyhydramnios (AFI ≥ 24 cm), oligohydramnios (AFI ≤ 5 cm). The gestational age of pregnancy at the time of delivery was considered as the gestational week for measurement. The analysis excluded multiple pregnancy, because in multiple pregnancy, chronicity is important for perinatal outcomes. Since the number of multiple pregnancies was minimal it would have been difficult to know the effect of chronicity on outcomes.

Patients having mild case polyhydramnios or oligohydramnios were rarely given intervention. However, with moderate and severe cases options like amniotic fluid drainage or pharmacological intervention, the patient’s consent and physician’s assessment of the scenario varied. Patients with gestational diabetes were given treatment to treat it which in many cases also helped in resolving polyhydramnios or oligohydramnios.

The statistical analysis was done using Statistical Package of Social Sciences (SPSS). Descriptive statistics in terms of frequency and percentages was used to describe criteria of studied sample. Association of qualitative variables was conducted by chi-square test. *P*-value less than 0.05 will be considered as statistically significant. Confidentiality of the collected data was ensured, and the information was only used for scientific research purposes. The methodology adhered to the STROBE guidelines which can be seen in supplementary Table 1.

## Results

Medical records of 597 patients were reviewed in this study, among them 453 (91.1%) were Saudis, around 388 (78%) were ≤ 35 years old. About 288 (57.9%) of the maternal BMI scores were ≥ 30. Around 124 (24.9%) of the patients were primigravida, however 373 (75.1%) of them were multigravida. Chronic medical illnesses were reported around 74 (14.8%). These patients were also getting adequate treatment for their chronic diseases, specifically in cases when there’s high risk of pregnancy or mother’s health being affected. Majority of the cases i.e., 425 (85.5%) had normal amniotic fluid index (AFI). However, 14 (2.8%) had polyhydramnios, and 58 (11.7%) had oligohydramnios (Table 1).

**Table 1:** Demographic Data of the Mothers (Frequency)

***Variable***	***Frequency*** ***(N)***	***Percentage*** ***(%)***
***Nationality***		
Saudi	453	91.1%
Non-Saudi	44	8.9%
***Age***		
≤ 35 (less than or equal 35)	388	78%
> 35 (more than 35)	109	22%
***Years of Education***		
School	2	0.4%
University	14	2.8%
Unknown	481	96.8%
***Work status***		
Housewife	7	1.4%
Employee	14	2.8%
Student	12	2.4%
Unknown	464	93.4%
***BMI***		
< 25 (normal and underweight)	48	9.7%
25–29.9 (overweight)	161	32.4%
≥ 30 (obese)	288	57.9%
***Gravity***		
Primigravida	124	24.9%
Multigravida	373	75.1%
***Chronic Medical Illness***		
Hypertension	3	0.6%
Diabetes mellitus	8	1.6%
Heart disease	2	0.4%
Renal disease	0	0.0%
Bronchial asthma	16	3.2%
Hypothyroidism	45	8.9%
***Amniotic Fluid Index***		
Normal	425	85.5%
Polyhydramnios	14	2.8%
Oligohydramnios	58	11.7%

The results (Table 2) showed that almost half of the neonate were boys 246 (49.5%), and the other half were girls 251 (50.5%). Only one case still born was identified 0.2%, while 496 (99.8%) were born alive. Majority of the patients were delivered at term 424 (85.3%), whereas, other 31 (6.2%) and 42 (8.5%) had a preterm and late term delivery respectively. The APGAR score of < 7 at 1 min and 5 mins interval was found in 3 and 2 children respectively. About 405 (81.6%) of them were admitted to nursery and the rest 91 (18.3%) were admitted to NICU. While, the congenital anomalies were diagnosed in 31(6.3%) of neonates.

**Table 2:** Frequency of Neonatal Outcome

Variable	Frequency	Frequency (%)
***Gender***		
Girl	251	50.5%
Boy	246	49.5%
Total	497	100%
***Living status***		
Live born	496	99.8%
Still born	1	0.2%
Neonatal death	0	0.0%
Total	497	100%
***Gestational age***		
Preterm < 37 weeks	31	6.2%
Term ≥37–41 weeks	424	85.3%
Late term ≥42 weeks	42	8.5%
***Admission of baby***		
Nursery	405	81.6%
NICU	91	18.3%
***Congenital anomalies***		
Yes	31	6.3%
No	465	93.7%
Total	496	100%
^a^***APGAR score***		
< 7 at 1 min	3	0.6%
< 7 at 5 min	2	0.4%

^a^missing 4 cases

There is no significant association between maternal age and the occurrence of amniotic fluid disorders. Amniotic fluid disorders found to be higher in multigravida and BMI group ≥30 with non-significant *P*-value. A statically significant association was found between gestational age at delivery and amniotic fluid disorders, polyhydramnios with late term deliveries. Besides, the rate of term deliveries was equal in normal AFI and oligohydramnios group (*P* = 0.005). However, the rate of caesarean section (CS) was higher among pregnancies complicated with polyhydramnios, and oligohydramnios was found to be majorly associated with vaginal deliveries (*P* = 0.008). Oligohydramnios was associated with neonatal birth weight < 2.5 kg and > 2.5 kg, and polyhydramnios was associated with neonatal birth weight ≥ 2.5 Kg (*P* < 0.01). Cardiovascular and renal congenital anomalies were diagnosed in neonates with polyhydramnios and oligohydramnios respectively with a non-significant *P*-value. (Table 3).

**Table 3:** Association Between the Types of Amniotic Fluids

		Normal AFI N (%)	Polyhydramnios N (%)	Oligohydramnios N (%)	***P***- value
***Maternal age***	≤ 35	329 (77.4%)	9 (64.3%)	50(86.2%)	0.14
	> 35	96 (22.6%)	5 (35.7%)	8 (13.8%)	
***Maternal parity***	Primigravida	105 (24.7%)	3 (21.4%)	16 (27.6%)	0.85
	Multigravida	320 (75.3%)	11 (78.6%)	42 (72.4%)	
***BMI***	< 25	44 (10.3%)	0 (0.0%)	4 (6.9%)	0.76
	25–29.9	138 (32.5%)	4 (28.6%)	19 (32.8%)	
	≥30	243 (57.2)	10 (71.4%)	35 (60.3%)	
***Gestational age at delivery***	Preterm < 37 weeks	21 (5.0%)	2 (14.3%)	8 (13.8%)	0.005
	Term 37–41 weeks	366 (86.1%)	9 (64.3%)	49 (84.5%)	
	Late term ≥42	38 (8.9%)	3 (21.4%)	1 (1.7%)	
***Admission of baby***	Nursery 405	352 (83.0%)	9 (64.3%)	44 (75.9%)	0.11
	NICU 91	72 (17.0%)	5 (35.7%)	14 (24.1%)	
***Mode of delivery***	Vaginal	256 (60.2%)	2 (14.3%)	36 (62.1%)	0.008
	Instrumental	19 (4.5%)	2 (14.3%)	2 (3.4%)	
	Emergency CS	78 (18.4%)	5 (35.7%)	14 (24.1%)	
	Elective CS	72 (16.9%)	5 (35.7%)	6 (10.3%)	
***Neonatal birth weight***	< 1 kg	4 (0.9%)	0 (0.0%)	1 (1.7%)	< 0.01
	1–1.4 kg	10 (2.4%)	0 (0.0%)	4 (6.9%)	
	1.5–2.4 kg	19 (4.5%)	0 (0.0%)	10 (17.2%)	
	2.5 - 4 kg	365 (86%)	12 (85.7%)	38 (65.5%)	
	> 4 kg	26 (61.3%)	2 (14.3%)	5 (8.6%)	
***Congenital malformation***	No anomaly	398 (93.8%)	12 (85.7%)	55 (94.8%)	0.16
	Cardiovascular	11 (2.6%)	2 (14.3%)	0 (0.0%)	
	Neurological	1 (0.2%)	0 (0.0%)	0 (0.0%)	
	Renal	14 (3.3%)	0 (0.0%)	3 (5.2%)	

Results in Table 4 indicate a remarkable significant association between oligo and polyhydramnios and patients with diabetes mellitus (*P* = 0.005), and gestational diabetes patients (*P* = 0.052). However, there was no significant association between oligo or polyhydramnios and hypertension, heart disease, bronchial asthma, hypothyroidism and anemia.

**Table 4:** Association Between Past Maternal History or chronic illness with Polyhydramnios and Oligohydramnios

Past Medical History	Normal AFI N (%)	Polyhydramnios N (%)	Oligohydramnios N (%)	***P***-value
Hypertension	2 (0.5%)	1 (7.1%)	0 (0%)	0.116
Diabetes mellitus	4 (0.9%)	2 (14.3%)	2 (3.4%)	0.005
Heart disease	2 (0.5%)	0 (0%)	0 (0%)	1
Bronchial asthma	13 (3%)	1 (7.1%)	2 (3.4%)	0.426
Gestational Diabetes mellitus	43 (10.1%)	4 (28.6%)	4 (6.9%)	0.052
Hypothyroidism	38 (8.9%)	1 (7.1%)	6 (10.3%)	0.902
Anemia	14 (3.3%)	1 (7.1%)	1 (1.7%)	0.517
Other	40 (9.4%)	2 (14.3%)	1 (1.7%)	0.072

## Discussion

The above results were based on the data obtained from the electronic medical records of 597 patients admitted in the department of obstetrics and gynecology at KAAUH, during January 2017 to October 2019. According to the results, 14.8% of the sample had different chronic medical illness, where 75.1% were multigravida. Besides, 14 (2.8%) of the sample were diagnosed as polyhydramnios and 58 (11.7%) of the cases were oligohydramnios based on their amniotic fluid index. Regarding the mode of delivery, 10 (71.4%) of polyhydramnios cases required caesarean section, and almost all cases of abnormal amniotic fluids showed no significant association with congenital malformation. The most significant maternal risk factors associated with amniotic fluid disorders include diabetes mellitus and gestational diabetes.

Majority of the mothers had live born neonates, and only single case (0.2%) of stillbirth was documented with no obvious reason due to the lack of data. Similarly, a study conducted at King Abdul-Aziz University Hospital in Jeddah showed that 5356 out of 5432 patients enrolled in the study had liveborn neonates, which represented 98.6% [7], this difference in percentage might be due to the large sample size enrolled in their study in comparison to the enrolled sample used in this research. More than 20% neonates of total deliveries got admitted in NICU due to various medical reasons such as lower APGAR score or congenital anomalies, out of which 18.3% of 496 liveborn neonates were admitted to NICU that were included in this study. Here, 6.2% of total babies were diagnosed with congenital anomalies, 22.6% of them were cardiovascular, while 54.8% were renal. Although, no relation was established with amniotic fluid disorders, the percentage is significantly higher compared to the previous study where 160 out of 5432 (2.9%) babies were diagnosed with congenital malformations [7].

In the present study, the maternal age was divided into two age groups i.e. participants less or equal to 35 years, and participants with above 35 years of age. However, most of the study participants (77.4%) were below 35 years, and the occurrence of amniotic fluid disorders was the same compared to the other age group (22.6%) of above or equal to 35 years. Thus, no association was found between maternal age and amniotic fluid disorders. Corresponding to another study done in Ondokuz Mayis University, Turkey, maternal age has no effect on perinatal and neonatal outcomes [8].

A statically significant association was found between gestational age at delivery and amniotic fluid disorders, polyhydramnios cases were mostly associated with late term deliveries which defined as a delivery after 42 weeks of gestation (*P* = 0.005), while the rate of term deliveries was equal in normal AFI and oligohydramnios group. These results are in contrast with another research conducted in Spain, which stated that oligohydramnios and polyhydramnios cases were associated with shorter gestational age [9] It could be due to the mild cases of polyhydramnios or successful treatment that the mothers underwent.

Among our collected sample, the rate of cesarean deliveries was higher in polyhydramnios cases comparing to normal cases, as mentioned by Yefet & Daniel-Spiegel [10] that, “This was attributed to an increased rate of elective surgeries because of suspected macrosomia”. In addition, the rate of vaginal deliveries was greater among oligohydramnios cases. These results are in contrast with those proposed by a research conducted in India which stated that the rate of vaginal deliveries was second highest after cesarean deliveries [11]. Also, 14 cases of polyhydramnios and 43 cases of oligohydramnios neonates weighted more than or equal to 2.5 Kg, whereas, 15 (25.9%) cases of oligohydramnios neonates were 2.5 Kg or less than it. A highly considerable association was found between polyhydramnios cases and neonates who weighted ≥2.5 kg. However, most of the neonates who weighted < 2.5 kg were diagnosed as oligohydramnios. Same association was found in a previous research which proved that the amniotic fluid disorders are mostly associated with lower birth weight [9].

No obvious relation was established between high body mass index (BMI above 25) and amniotic fluid disorders. Besides, more than half of the sample were obese 57.9, and 32.4% were overweight. Correspondingly, another research conducted in 2016 revealed that KSA is considered as the 15th most obese country with rate of 33.7% among population, thus no relation can be established [12]. However, there were increased cases of poly and oligohydramnios and congenital anomalies in this study, this could be because majority of them are obese or overweight as maternal obesity is linked to pregnancy complications and neonatal outcomes [13].

It has been noted that the occurrence of polyhydramnios was highly related to chronic disease of diabetes mellitus with *P*-value equal to 0.005. Similar value was shown with mothers who suffered from gestational diabetes mellitus, as outlined in one of the relevant articles that the most notable maternal risk factor behind polyhydramnios is diabetes mellitus [3] which was also associated with the cases represented oligohydramnios. Hence, it can be said that past maternal history of diabetes mellitus is the main risk factor behind amniotic fluid disorders according to the research outcome. Other maternal chronic diseases showed no effect on amniotic fluid index although it might cause other risks on the fetus. Similarly, no association was found between hypertensive disorder of pregnancy and oligohydramnios, which could be due to the mild cases or treatment received by the mothers.

This study has important implications, as the findings may help medical experts in providing proper counselling regarding pregnancy related complications found among most of the Saudi women. It will further help medical experts to consider all the risk factors associated to the amniotic fluid disorders and its impact on maternal and fetal outcome. These findings may serve as highly useful in reducing the risks associated to the amniotic fluid disorders [14]. The study was limited as it was a single center study. Also, the effects of treatment for chronic conditions of mothers and its impact on the amniotic fluid disorder was not studied due to the availability of limited data.

## Conclusion

Findings of this study confirms that maternal risk factors which were associated with amniotic fluid disorders included diabetes mellitus and gestational diabetes. Regarding the maternal and fetal outcomes, the results indicated that oligohydramnios associated with gestational age at term and neonatal birth weighted < 2.5 kg, and polyhydramnios associated with gestational age at late term and neonatal birth weighted > 2.5 Kg. Regarding the mode of delivery, it was found that CS was higher among pregnancies which were complicated due to polyhydramnios while oligohydramnios was mostly associated with vaginal deliveries.

## Supplementary Information

**Additional file 1.** STROBE Statement—Checklist of items that should be included in reports of retrospective studies.

## Data Availability

The datasets used and analyzed during the current study are available from the corresponding author on reasonable request.

## Abbreviations

AFI: Amniotic Fluid Index
AFV: Amniotic Fluid Volume
BMI: Body Mass Index
CS: Cesarean Section
KAAUH: King Abdullah bin Abdul-Aziz University Hospital
NICU: Neonatal Intensive Care Unit
PNU: Princess Nourah bint Abdulrahman University
SDP: Single Deepest Pocket
SPSS: Statistical Package of Social Sciences
